# Blood-Borne Biomarkers of Mortality Risk: Systematic Review of Cohort Studies

**DOI:** 10.1371/journal.pone.0127550

**Published:** 2015-06-03

**Authors:** Evelyn Barron, Jose Lara, Martin White, John C. Mathers

**Affiliations:** 1 Human Nutrition Research Centre, Institute of Cellular Medicine, Newcastle University, Biomedical Research Building, Campus for Ageing & Vitality, Newcastle upon Tyne NE4 5PL, United Kingdom; 2 Institute of Health and Society, Newcastle University, Baddiley-Clark Building, Faculty of Medical Sciences, Newcastle upon Tyne, NE2 4AX, United Kingdom; 3 Fuse, UKCRC Centre for Translational Research in Public Health, Institute of Health & Society, Baddiley-Clark Building, Faculty of Medical Sciences, Newcastle upon Tyne, NE2 4AX, United Kingdom; Universidad Europea de Madrid, SPAIN

## Abstract

**Background:**

Lifespan and the proportion of older people in the population are increasing, with far reaching consequences for the social, political and economic landscape. Unless accompanied by an increase in health span, increases in age-related diseases will increase the burden on health care resources. Intervention studies to enhance healthy ageing need appropriate outcome measures, such as blood-borne biomarkers, which are easily obtainable, cost-effective, and widely accepted. To date there have been no systematic reviews of blood-borne biomarkers of mortality.

**Aim:**

To conduct a systematic review to identify available blood-borne biomarkers of mortality that can be used to predict healthy ageing post-retirement.

**Methods:**

Four databases (Medline, Embase, Scopus, Web of Science) were searched. We included prospective cohort studies with a minimum of two years follow up and data available for participants with a mean age of 50 to 75 years at baseline.

**Results:**

From a total of 11,555 studies identified in initial searches, 23 fulfilled the inclusion criteria. Fifty-one blood borne biomarkers potentially predictive of mortality risk were identified. In total, 20 biomarkers were associated with mortality risk. Meta-analyses of mortality risk showed significant associations with C-reactive protein (Hazard ratios for all-cause mortality 1.42, p<0.001; Cancer-mortality 1.62, p<0.009; CVD-mortality 1.31, p = 0.033), N Terminal-pro brain natriuretic peptide (Hazard ratios for all-cause mortality 1.43, p<0.001; CHD-mortality 1.58, p<0.001; CVD-mortality 1.67, p<0.001) and white blood cell count (Hazard ratios for all-cause mortality 1.36, p = 0.001). There was also evidence that brain natriuretic peptide, cholesterol fractions, erythrocyte sedimentation rate, fibrinogen, granulocytes, homocysteine, intercellular adhesion molecule-1, neutrophils, osteoprotegerin, procollagen type III aminoterminal peptide, serum uric acid, soluble urokinase plasminogen activator receptor, tissue inhibitor of metalloproteinases 1 and tumour necrosis factor receptor II may predict mortality risk. There was equivocal evidence for the utility of 14 biomarkers and no association with mortality risk for CD40 ligand, cortisol, dehydroepiandrosterone, ferritin, haemoglobin, interleukin-12, monocyte chemoattractant protein 1, matrix metalloproteinase 9, myelopereoxidase, P-selectin, receptor activator of nuclear factor KappaB ligand, sex hormone binding globulin, testosterone, transferrin, and thyroid stimulating hormone and thyroxine.

**Conclusions:**

Twenty biomarkers should be prioritised as potential predictors of mortality in future studies. More studies using standardised protocols and reporting methods, and which focus on mortality rather than risk of disease or health status as an outcome, are needed.

## Introduction

Population demographics are changing worldwide, as people are living longer and the birth-rate is falling in many countries. In the UK the number of adults over 60 years of age is expected to increase by 7 million over the next 20 years [1]. The proportion of the oldest old is also growing with a predicted sevenfold increase in the number of centenarians by the middle of this century [2]. This increase in lifespan has been attributed to a combination of factors, including higher standards of living, education, prosperity, improved healthcare and healthier lifestyles [3]. However, increased lifespan does not necessarily equate to increased years of good health and can often mean a longer period of morbidity before death [4]. Longer lives and increased age-related frailty, disability and disease will have far reaching consequences for the social, political and economic landscape. Unless increased lifespan is accompanied by increased health span, the greater burden of age-related disease will put pressure on health care resources [4, 5] and require changes in the way these resources are distributed [6].

Research is beginning to focus on interventions to help health span to keep pace with lifespan. Most interventions focus on lifestyle factors including physical activity and diet (e.g. [7] as means to slow functional decline, improve mood, general health (e.g. [8], cognitive performance [9, 10] and lower mortality rates [11]. However, to assess the impact of such interventions, outcome measures are needed which are suitable for use in large community based studies and which are cost-effective, easily obtainable, and sufficiently sensitive to detect change in response to interventions [12].

Biomarkers are objective measures that may be useful for assessing the utility of such interventions. Biomarkers of ageing should be better predictors of functional change than chronological age alone [13]. In addition Johnson has proposed that a biomarker of ageing must monitor a process of ageing, not an underlying disease process; be usable repeatedly without causing harm; and work equally well in animal models so that they can be validated before being used in humans [14]. Blood-borne biomarkers fulfil these criteria [15] and are potentially appropriate outcome measures for intervention studies. Despite decades of research, there are few reliable markers of ageing processes and questions remain over the reliability and validity of such biomarkers [16, 17]. It is difficult to assess the concurrent validity of biomarkers because there is no gold-standard measure of healthy ageing [18], so for this reason we chose mortality as the outcome in this analysis.

The aim of this systematic review was to identify blood-borne biomarkers predictive of mortality which could potentially be used to assess the utility of interventions designed to improve health span. Evidence from studies of physical activity and diet strongly suggest that the retirement transition is an ideal target for lifestyle based interventions to promote healthy ageing (e.g. [19, 20]. Therefore, this systematic review focused on studies with participants in the retirement transition age window, during which interventions to promote health and wellbeing in later life could be delivered.

## Methods

### Protocol

The review was conducted according to established methods (Cochrane, CRD [21]) and is reported according to PRISMA guidelines [22]. The protocol was registered with the PROSPERO database (Prospective Register of Systematic Reviews, Registration number: CRD42011001499).

### Selection Criteria

#### Study type

Studies with a prospective cohort design and at least 2 years follow up were included to allow the predictive value of biomarkers to be assessed. Where studies investigated disease prevention, only primary prevention studies were included. For Medline and Embase, standardised filters from BMJ Evidence [23] which return cohort studies were used. Equivalent versions were not available for Web of Science and Scopus, so topic searching and index terms respectively were used to search for cohort studies. Searches were conducted up to March 2014.

#### Participants

Studies that included human participants, of either sex, with blood-borne biomarker data available for participants with a mean age of 50–75 years at baseline were included. Studies that recruited participants based on risk factors for a disease, rather than presence of the disease itself, were included as these studies were likely to focus on primary prevention.

#### Outcome measures

This review was restricted to studies examining biomarkers that are blood-borne and where a relationship with mortality was reported. Studies that examined genetic factors or biomarkers diagnostic of a particular disease rather than mortality risk were not included in this review.

### Search Strategy

#### Search Terms

A systematic review of the literature was conducted across Medline, Embase, Scopus and Web of Science. The search strategy was created in Medline and translated for the three other databases. The specific search strategy (S1 File) included ‘biological marker’ and ‘marker’, combined with ‘blood’ and four alternative terms: ‘plasma’, ‘serum’, ‘DNA’ and ‘circulating’ and relevant synonyms. Age filters were used to increase the specificity of the search. Reference lists of identified articles were hand searched for further relevant articles.

#### Publication screening, data extraction and quality assessment

Two investigators (EB and JL) conducted title and abstract screening to identify articles justifying full text screening according to the selection criteria. Any disagreements were resolved by discussion. The reference lists of studies accepted after full text screening were cross-checked by hand to identify other relevant publications. Studies identified from reference cross-checking were subjected to the same process. Data were extracted from the full text article of all studies accepted after full text screening using a customised data extraction form (S2 File). The data extraction form was developed based on the York CRD guidelines [21] and the STROBE (Strengthening the Reporting of Observational Studies in Epidemiology) tool [24]. The form was piloted on a sample of studies to be included in the review. Quality was assessed using the Newcastle-Ottawa Scale [25].

### Statistical data analysis

Comprehensive meta-analysis (Version 2, Biostat, Englewood NJ) and Stata (Stata/SE 11.2 for Windows; StataCorp LP, College Station, Texas, USA) were used to pool and to meta-analyse results from the individual studies. Pooled results were extracted and analysed as Hazard Ratios (HR) with 95% confidence intervals. Hazard ratios for biomarkers were analysed by subgroup, where possible. Overall Hazard ratios are also presented. A random-effects model was used and 2-sided P values from an inverse variance statistical method are reported.

Statistical heterogeneity was evaluated using the I^2^ statistic [26, 27], which assesses the appropriateness of pooling individual study results and the 95% CI for I^2^ were calculated using Higgins et al’s method [28, 29]. Where I^2^ was >50%, the degree of heterogeneity was considered substantial. Potential publication bias was appraised by visual inspection of the funnel plot of effect size against the SE for each study, with asymmetry formally assessed with Egger’s regression test.

## Results

### Search Results

**Fig 1:**
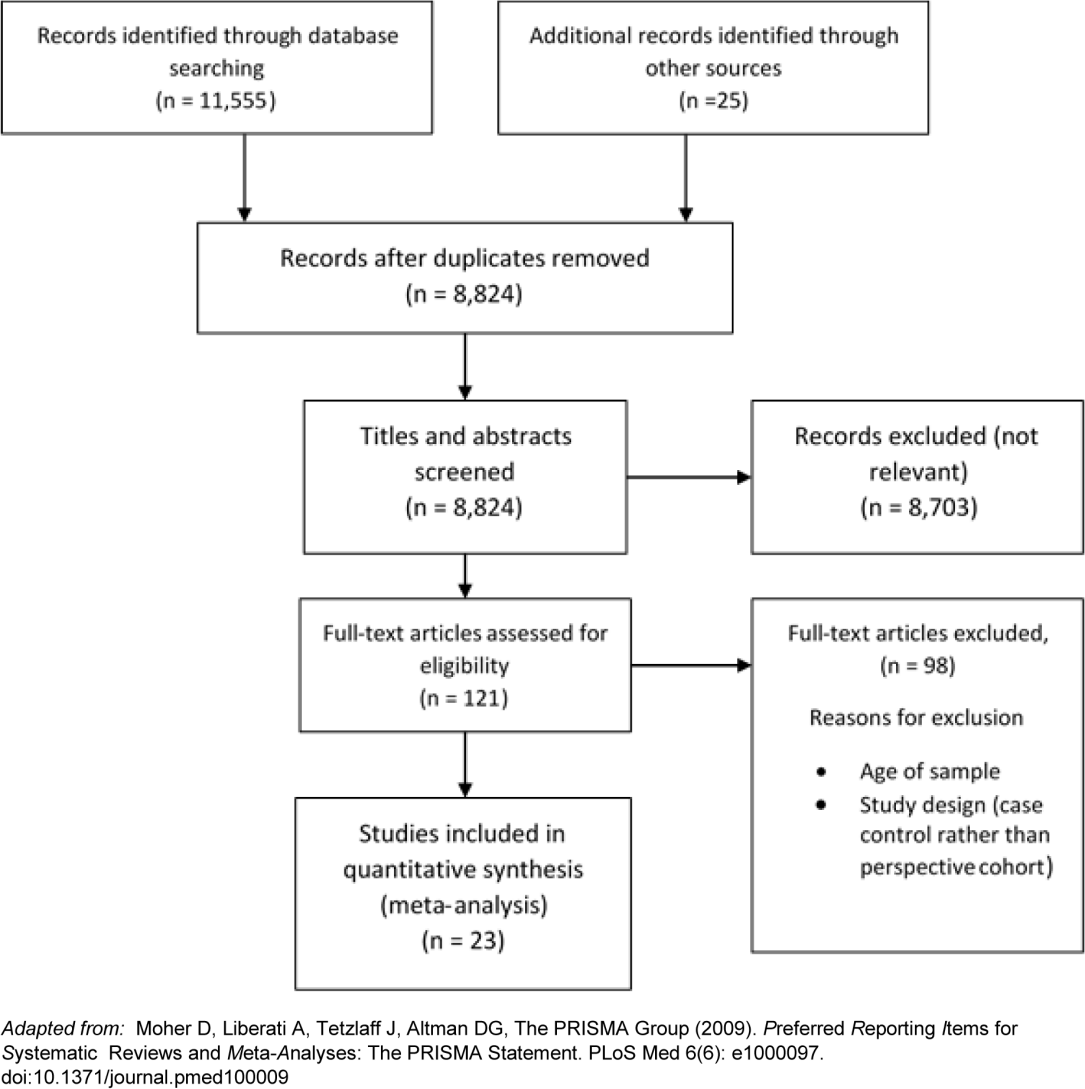
PRISMA diagram. Prisma diagram showing the number of references identified in the search and the number of inclusions and exclusions at each stage.

Fig 1 summarises the number of papers at each stage of the search. The initial search returned 11,555 papers for possible inclusion in the review. After title and abstract screening, 8,703 papers did not meet selection criteria. One hundred and twenty one studies were screened as full-text, and 21 studies meeting our inclusion criteria were included in this review. After screening the references of these studies, two more publications meeting our inclusion criteria were identified [30–52]. All studies were of a similar quality according to the NOS [25].

#### Study characteristics

The characteristics of the participants in the cohorts included in the review varied widely, as did sample size (range: 254 to 160,309 participants), length of follow up (range: 2.7 to 19 years) and gender balance (see Table 1). The majority of studies were from Europe (11) with nine studies from the USA, two studies from Japan and one from Korea.

**Table 1:** Study characteristics.

F	Location	Cohort	Enrolment period	Sample size	Mean length of follow up (years)	Age	Sex		Biomarker (Interquartile range of concentration or mean (SD))	Type of mortality	Confounders adjusted for
						Mean±SD (Range)	Male	Female			
**Baune et al (2011)[31]**	Germany	MEMO	1997–1998	385	9	72.7±N/A (65–83)	195 (53%)	174 (47%)	Il-1b (pg/ml) (-)Il4sR (pg/ml) (-)Il-6 (pg/ml) (<0.68–2,19)Il-8 (pg/ml) (<5.58–8.39)Il-10 (pg/ml) (<1.41–2.97)Il-12 (pg/ml) (-)TNFa (ng/ml) (-)	All-cause	Age, sex, smoking status
**Baylis et al (2013)[42]**	UK	Hertfordshire Ageing Study	1994–1995	254	10	Males 66.9±2.2Females 67.3±2.1	153 (60%)	101 (40%)	WBC count (x10^9^/L) (male 4.8–6.6, female 4.7–6.5) ESR (mm/hr) (male 4–10, female 8–20) Neutrophils (x10^9^/L) (male 2.8–4, female 2.5–4) Monocytes (x10^9^/L) (male 0.3–0.5, female 0.3–0.4)Lymphocytes (x10^9^/L) (male 1.32–2, female 11.4–2.1) Albumin (g/L) (male 42.7 (2.0) female 42.3 (2.0))SHBG (nmol/L) (male 27.9–48.3, female 34.2–72.9) Testosterone (nmol/L) (male 11.1–20.3)Haemoglobin (g/L) (male 14.5 (1) female 13.5 (0.9)) TSH (mU/L) (male 1.2–2.3, female 1.5–3.3) T4 (pmol/L) (male 13.2–15.6, female 12.6–15.3) IL-1b (pg/mL) (male 5.6–17.7 female 11.7–26.2) IL-6 (pg/mL) (male 0.2–2.0 female 0.4–2.0) IL-10 (pg/mL) (male 2.5–4.7 female 0.3–2.1) CRP (mg/L) (male 0.9–4.0 female 1.1–5.6) DHEAS (nmol/L) (male 1734–2888 female 797–2261) Cortisol (nmol/L) (male 235–285 female 210–349) Cortisol:DHEAS ratio (male 0.09–0.17 female 0.1–0.38)	All-cause	Age, alcohol, intake, BMI, comorbidity, height, sex, social class, walking speed
**Carriere et al (2008)[30]**	France	Pathologies Oculaires Liées à l’Age	1995–1997	1,441	9	70±6.6 (60+)	553 (38%)	888 (62%)	TC (mmol/L) (male <4.95–6.18 female <5.23–6.61) Albumin (g/L) (male <39.44–44.78 female <38.93–43.94) TTR (g/L) (male <0.24–0.3 female <0.21–0.27) CRP (mg/L) (male <0.86–3.31 female <0.79–3.27) AAG (g/L) (<0.61–0.90)	All-cause Cancer CVD	Educational level, perceived health, smoking (current)
**Cui et al (2001)[32]**	USA	Lipid Research Clinics Program Follow-up Study	1972–1976	4,462	19	50.1±6.6 (40–64)	2,406 (54%)	2,056 (46%)	Non HDL-C (mmol/L & mg/dL) (male 4.69 (1.22) female 4.45 (1.41)) HDL-C (mmol/L & mg/dL) (male 1.16 (0.33) female 1.54 (0.45)) LDL-C (mmol/L & mg/dL) 3.85 (1.05) female 3.89 (1.16)	All-cause CVD	Age, alcohol intake, body mass index, fasting glucose, hypertension, smoking
**Emerging Risk Factors Collaboration (2010)[43]**	UK	Meta-analysis of 54 studies	-	160,309	1.31 million person years at risk	60±8	84138 (52%)	76171 (48%)	CRP (mg/L) 1.11	Vascular and non-vascular mortality	Age, alcohol consumption, BMI, diabetes, HDL cholesterol, non-HDL cholesterol, sex, study, systolic blood pressure, smoking, triglyceride concentration
**Eugen-Olsen et al (2010)[33]**	Denmark	MONICA10	1993–1994	2,602	13.6 (median)	56±-(41–71)	-	-	SUPAR (ng/mL) (1.3–19.9) CRP (mg/L) (<1–3)	All-cause Cancer CVD	Age, sex, smoking status
**Kabagambe et al (2011)[44]**	USA	REGARDS	2003–2007	17,845	4.52 (median)	65.6±-(45+)	6782 (38%)	11063 (62%)	CRP (mg/L) (1.28–12.47) WBC count (x10^9^cells/L) (5.09–8.21) Albumin (g/dL) (3.7–4.3)	All-cause	Age, alcohol use, educational level, HDL-C, income, LDL-C medical history, physical activity frequency race, region, sex, smoking, triglycerides and waist circumference
**Kim et al (2013)[41]**	Korea	-	2003–2009	9996	4.4 (median)	69.7±4.3(65+)	5491(54.9%)	4505(45.1%)	White blood cells (cells/μL) (<4670–6610)	All-cause Cancer CVD	Age, alcohol intake, alanine aminotransferase, albumin, aspartate aminotransferase, blood pressure, body mass index, diabetes mellitus, erythrocyte sedimentation rate, exercise levels, sex, GFR, HDL-cholesterol, non-HDL cholesterol, smoking status.
**Kistorp et al (2005)[34]**	Denmark	-	1998–2000	626	5	67.9±10.6 (50–89)	265 (43%)	361 (57%)	N-terminal pro-brain natriuretic peptide (pg/mL) (<181.7–411.1) CRP (mg/L) (2.04–2.81)	All-cause	ACE inhibitors, beta-blockers, calcium antagonists, diabetes, diuretics hypertension, ischemic heart disease, heart rate, left ventricular hypertrophy, left ventricular systolic dysfunction, serum creatinine, smoking, systolic blood pressure, total cholesterol
**Koenig et al (2008)[35]**	Germany	MONICA	1984–1995	3,620	7.1	57.9±8.1 (45–74)	3620 (100%)	0	CRP (mg/L) (<1–3)	All-cause Cancer CVD CHD	Age, alcohol intake, body mass index, diabetes, dyslipidemia, education, hypertension, physical activity, smoking, year of recruitment
**Lieb et al (2010)[45]**	USA	Framingham Offspring	1971	3,250	4.6	61±9-	1495 (46%)	1755 (54%)	RANK-L (pmol/L) 0.05 (0.02–0.14) OPG (pmol/L) 5.40 (4.45–6.49)	All-cause CVD	Age, diabetes mellitus, diastolic blood pressure CRP, hypertension treatment, lipid-lowering medication serum glucose, sex, smoking, systolic blood pressure, and total/HDL cholesterol.
**Margolis et al (2003)[52]**	USA	Women’s Health Initiative Observational Study	1994–1998	72242	6.1	63±7.3 (50–79)	0	72242 (100%)	WBC count (x10^9^ cells/L) (2.5–15.0)	All-cause CHD	Age, race/ethnicity, diabetes, hypertension, high cholesterol level, smoking status, body mass index, alcohol intake, physical activity, aspirin use, dietary fibre, fruit/vegetable intake, polyunsaturated/saturated fatty acid ratio, and prior use of hormone therapy.
**Makita et al (2009)[36]**	Japan	Iwate-Kenpoku Cohort	2002–2005	7,901	2.7	64±9.7 (40–80)	7901 (100%)	0	C-reactive protein (mg/L) (0.1 >0.8)	All-cause	Age, body mass index, diabetes, glomerular filtration rate, high density lipoprotein cholesterol, smoking (current/past), systolic blood pressure, total cholesterol, uric acid
**McKie et al (2006)[37]**	USA	Rochester Epidemiology Project	-	1,991	5.6 (person years)	62±10 (45+)	952 (48%)	1,039 (52%)	N-terminal pro-brain natriuretic peptide (pg/mL) Brain natriuretic peptide (pg/mL)	All-cause	Age, coronary artery disease, diabetes, sex, hypertension serum creatinine, total cholesterol
**Menke et al (2012)[46]**	USA	NHANES III	1988–1994	12,258	Until 1996 or age 90, whichever came first	Presented by quartile	-	-	Ferritin (ng/mL) (males 87–222, females 18–158) Transferrin saturation (%) (males 21–35 females 15–31)	All-cause Cancer CVD	Age, alcohol consumption, aspirin, BMI, cholesterol-lowering medication, CRP, diabetes estimated glomerular filtration rate, ethnicity, HDL cholesterol, high school education, household income, HRT, hypertensive medication, smoking status, systolic blood pressure, total cholesterol and vitamin C supplementation
**Niskanen et al (2004)[38]**	Finland	Kuopio Ischaemic Heart Disease Risk Factor Study	1984 & 1989	1,423	11.9	52.3±5.3 -	1423 (100%)	0	SUA (mm/dL) 5.67 (1.01)	All-cause CVD	Age, alcohol intake, beta-blockers, blood pressure medication, BMI, cardiovascular fitness, diuretic use, examination year, exercise, family history of coronary heart disease, fasting blood glucose, fasting serum insulin, high density lipoprotein cholesterol, low density lipoprotein cholesterol, serum creatinine, socioeconomic status, smoking, systolic blood pressure, triglycerides
**Okamura et al (2006)[39]**	Japan	NIPPON	1990	7,175	9.6	Males 52.8±13.5 Females 51.8±13.8 (30+)	3014 (42%)	4161 (58%)	HDL-C (mmol/L) (1.04–1.56)	All-cause Cancer CVD	Age, alcohol intake, body mass index, cholesterol, diabetes, sex, hypertension, smoking triglycerides
**Strasak et al (2008)[40]**	Austria	Vorarlberg Health Monitoring and Promotion Program	1985–2005	28,613	15.2 (median)	62.3±8.8 (50–95.3)	0	28,613 (100%)	Serum uric acid (mg/dL) 4.6 (1.3)	CVD	Age, blood pressure, gamma-glutamyl-transferase, total cholesterol, triglycerides
**Schnabel et al (2013)[47]**	USA	Framingham Heart Study	-	3,035	8.9 (median) 11.3 (maximum)	61±9-	1412 (47%)	1623 (53%)	CRP 0.82(1.12) Fibrinogen 5.92 (0.19)IL-6 1.07 (0.71) ICAM-1 5.52 (0.25) La-PlA2: mass 5.65 (0.32), activity 493 (0.25) MCP-1 5.74 (0.34) Myeloperoxidase 3.71 (0.57) CD40 ligand 0.41(1.23) P-selectin 3.57 (0.73) TNFRII 7.62 (0.31)	All-cause CVD	Age, BMI, current smoking, diabetes mellitus, hypertension treatment. Sex, systolic blood pressure and total/high-density lipoprotein-cholesterol.
**Velagaleti et al (2010)[48]**	USA	Framingham Offspring	1971	922	9.9,12.7 (maximum)	58(10)-	406 (44%)	516 (56%)	MMP-9 (% detectable) 20 TIMP-1 (ng/mL) 20 (4.0) PIIINP (ng/mL) 4.0 (3.8)	All-cause CVD	Age, BMI, current smoking, diabetes mellitus, hypertension treatment, LVM and LV sampling group. Sex, systolic blood pressure, and total cholesterol/ high-density lipoprotein cholesterol ratio
**Wannamethee et al (2011)[49]**	UK	British Regional Heart Study	1978–1980	3,649	9	Mean age presented by quartile(60–79)	3,649 (100%)	0	CRP (-) NT-proBNP (pg/mL) (without CVD 40–151, with CVD 85–384)	CVD	Alcohol intake, anaemia, BMI, diabetes, eGFR, physical activity, smoking status and social class
**Welsh et al (2013)[50]**	UK	West of Scotland Coronary Prevention Study	(N/A)	6,595	14.7 (median)	-(45–65)	6595 (100%)	0	CRP (mg/L) 1.73 (4.60) NT-proBNP (pg/mL) 28.0 (61.0)	All-cause CVD	Age, BMI, C-reactive protein, diabetes, HDL and LDL cholesterol, history of angina, hypertension medication, nitrate use, smoking, social deprivation score systolic blood pressure, triglycerides,
**Wu et al (2011)[51]**	USA	NHANES III	1988–1994	10,245	13.2	Mean age presented by risk group(35+)	4873 (48%)	5372 (52%)	Creatinine μg/g (-) CRP (mg/dL) (-) Fibrinogen (mg/dl) (-) Cystatin C (mg/dl) (-) Uric acid (mg/dl) (-) 25(OH)D (ng/mL) (-) Homocysteine (umol/l) (-)	All-cause Cancer CVD	Age, blood pressure, cholesterol, diabetes, high- and low-density lipoprotein, smoking and triglycerides

- = data not available

Table 1 summarises the biomarkers examined and the mortality measure reported in each study. Fifty-one potential biomarkers of mortality were identified in this review: 25 hydroxyvitamin D, albumin, alpha 1-acid glycoprotein, brain natriuretic peptide, CD40 ligand, C-reactive protein, cortisol, creatinine, cystatin C, dehydroepiandrosterone sulphate, erythrocyte sedimentation rate, ferritin, fibrinogen, granulocytes, haemoglobin, high density lipoprotein cholesterol, homocysteine, intercellular adhesion molecule-1, interleukin 1β, interleukin 4 soluble receptor, interleukin-6, interleukin 8, interleukin 10, interleukin 12, lipoprotein associated phospholipase A2, low density lipoprotein cholesterol, lymphocytes, matrix metalloproteinase 9, myeloperoxidase, monocyte chemoattractant protein 1, monocytes, neutrophils, non-high density lipoprotein cholesterol, N-terminal pro-brain natriuretic peptide, osteoprotegerin, P-selectin, procollagen type III aminoterminal peptide, receptor activator of nuclear factor-kappaB ligand, serum uric acid, sex hormone binding globulin, soluble urokinase plasminogen activator receptor, T4, testosterone, thyroid stimulating hormone, tissue inhibitors of metalloproteinases 1, total cholesterol, transferrin, transthyretin, tumour necrosis factor alpha, tumour necrosis factor receptor II and white blood cell count. It was not possible to divide all of the analyses by sex as some of the original studies did not provide data for males and females separately.

### Meta-analyses

#### C-reactive protein (CRP)

**Fig 2:**
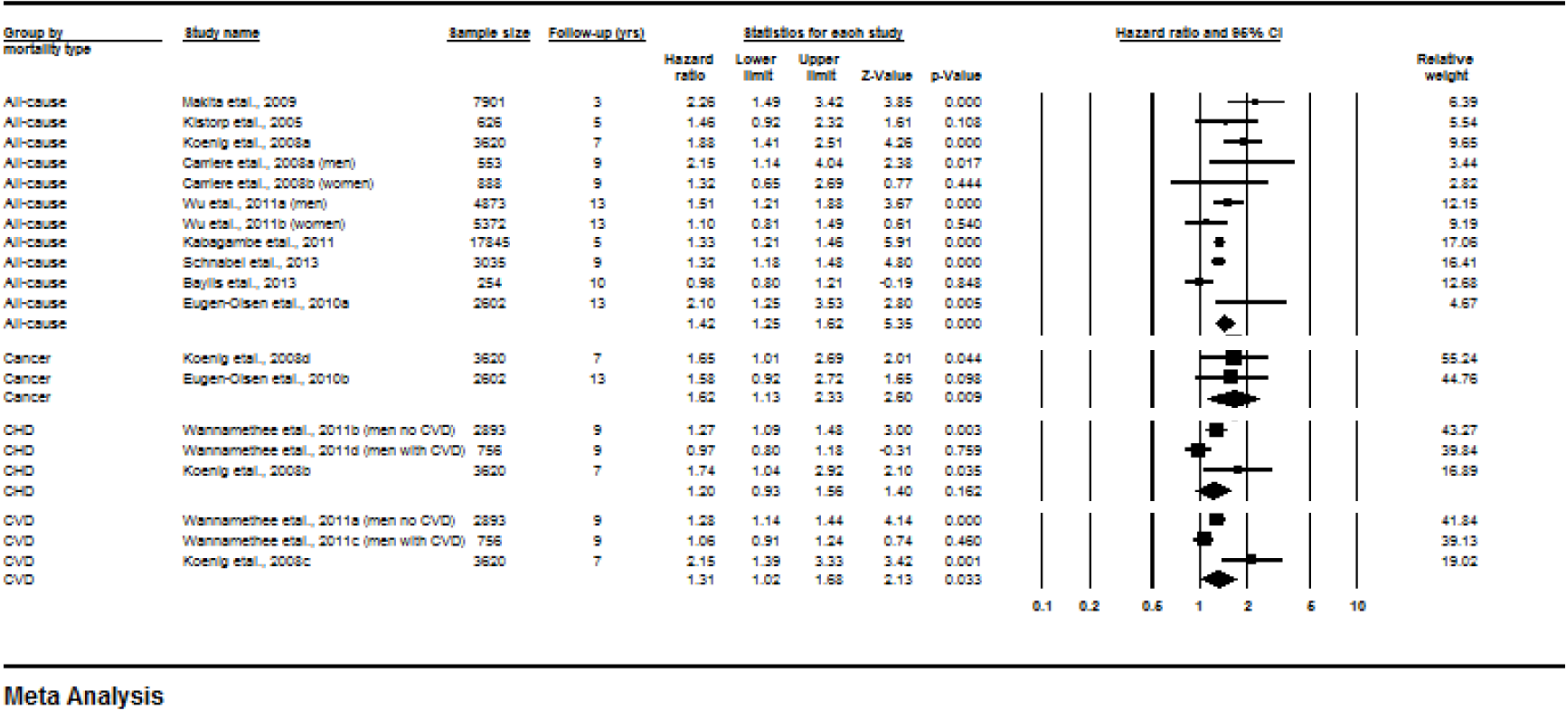
Forest plot of Hazard ratios for all-cause, cancer, CHD-related, and CVD-related mortality for each 1-SD increase in CRP.

C-reactive protein was examined in 12 studies [30, 33–36, 42–44, 47, 49–51]. Meta-analysis was conducted on the relationship between CRP and mortality and Fig 2 presents results by type of mortality. Higher CRP at baseline was significantly associated with an increased risk of all-cause mortality (HR 1.42, 1.25–1.62, p<0.0001; I^2^ = 64.38; Q 28.1; DF 10; p = 0.002) and cardiovascular disease (CVD) mortality (HR 1.31, 1.02–1.68, p = 0.033; I^2^ = 80.44; Q 10.22; DF 2; p = 0.006), but baseline CRP has no relationship with risk of coronary heart disease (CHD) mortality (HR 1.20, 0.93–1.56, p = 0.162; I^2^ = 71.02; Q 6.9; DF 2; p = 0.032). Regarding other specific causes of mortality, higher CRP concentrations at baseline were associated with greater risk of cancer mortality (HR 1.62, 1.13–2.33, p = 0.009). A funnel plot of all meta-analysed studies on CRP did not show asymmetry and Egger’s regression test was not significant (p = 0.44) indicating likely absence of publication bias.

Subgroup analysis by follow-up length showed that among studies with follow up of 5 years or less (HR, 1.57, 1.14–2.17, p = 0.006; I^2^ = 66.81; Q 6.02; DF 2; p = 0.049) and studies with follow-ups over 5 years (HR 1.40, 1.17–1.67, p<0.0001; I^2^ = 67.92; Q 21.81; DF 7; p = 0.003) the association between CRP and all-cause mortality remained significant. Comparison of these subgroups showed no significant differences (p = 0.540).

#### N-Terminal pro Brain Natriuretic Peptide (NT-proBNP)

**Fig 3:**
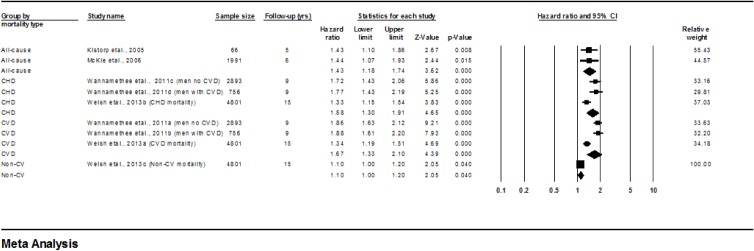
Forest plot of Hazard ratios for all-cause, CHD-related, CVD-related, and Non-CVD-related mortality for each 1-SD increase in NT proBNP.

Overall, higher concentrations of NT-proBNP at baseline were associated with greater subsequent mortality (Fig 3). Kistorp et al [34] and McKie et al [37] examined relationships between NT-proBNP and all-cause mortality risk while Wannamethee et al [49] and Welsh et al [50] examined links with CVD and CHD mortality. Higher concentrations of NT-proBNP were associated with greater risk of all-cause mortality (HR 1.43, 1.18–1.74, p<0.0001; I^2^ = 0; Q 0.001; DF 1; p = 0.97), CHD mortality (HR 1.58, 1.30–1.91, p<0.0001; I^2^ = 71; Q 6.93; DF 2; P = 0.031) and CVD mortality (HR 1.67, 1.33–2.10, p<0.0001; I^2^ = 88; Q 16.88; DF 2; p = 0.0002). One study reported an association with non-CVD mortality.

#### Interleukin-6 (IL-6)

**Fig 4:**
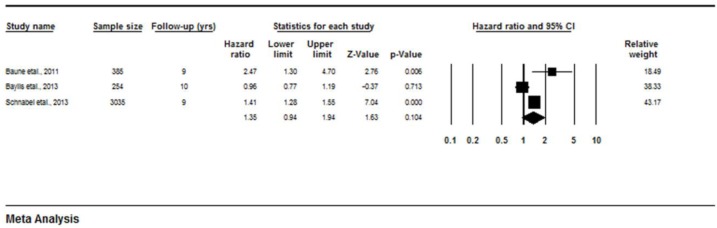
Forest plot of Hazard ratios for all-cause mortality risk for each 1-SD increase in IL-6.

Baune et al [31], Baylis et al [42] and Schnabel et al [47] investigated associations between IL-6 and mortality. Both Baune et al [31] and Schnabel et al [47] found significant associations between increased levels of IL-6 and increased mortality risk (HR 2.47, 1.3–4.7, p = 0.006; HR 1.41, 1.28–1.55, p<0.0001 respectively). However, Baylis et al [42] found no association (Fig 4) between IL-6 and 10 year mortality (HR 0.96, 0.77–0.91, p = 0.713). Overall, the meta-analysis showed no relationship between IL-6 and mortality risk (HR 1.35, 0.94–1.94, p = 0.104; I^2^ = 85.28 (95%CI 57 to 95); Q = 13.56, DF = 2; p = 0.0001).

#### White Blood Cell (WBC) count

**Fig 5:**
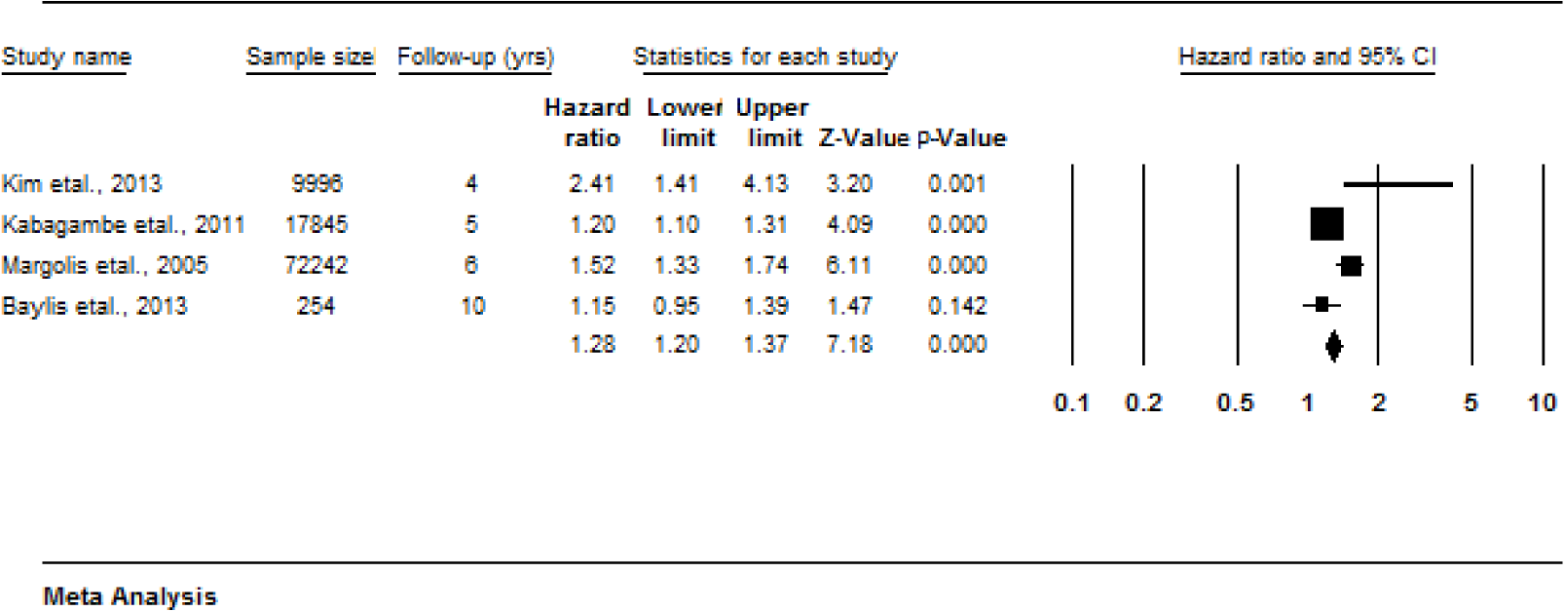
Forest plot of Hazard ratios for all-cause mortality risk and increases in white blood cell count.

Four studies examine the association between WBC count and all-cause mortality [41, 42, 44, 52]. Higher WBC count at baseline was associated with greater risk of all-cause mortality (Fig 5) (HR 1.36, 1.13–1.64, p = 0.001). I^2^ was significant (I^2^ = 79.9, (95%CI 47 to 92) Q = 14.95, df = 3, p = 0.0001). Although the Egger test was non-significant (p = 0.73), with only 4 studies the possibility of publication bias cannot be excluded.

### Narrative review

For the remaining putative biomarkers of mortality risk, there were too few studies to warrant meta-analysis.

#### 25 Hydroxyvitamin D (25(OH)D)

Overall, there was no association between 25(OH)D and all-cause, CV and non-CV mortality but higher 25(OH)D concentrations were protective in men with intermediate to high coronary risk scores for all-cause (HR 0.60, 0.44–0.82, p = 0.001) and CV mortality (HR 0.46, 0.29–0.73, p = 0.001) [51].

#### Brain Natriuretic Peptide (BNP)

There was a significant positive association between baseline BNP concentrations and all-cause mortality risk (HR 2.98,2.39–3.72, p<0.001) [37].

#### Carrier Proteins. Albumin

Carriere et al [30] examined the association between plasma albumin concentration and all-cause mortality risk at ≤5 and 5 to 9 years of follow up. Albumin concentrations in the lowest quartile were associated with higher mortality risk at ≤ 5 years follow up (HR 2.72, 1.44–5.14, p = 0.002) in males but no association was found in males at 5 to 9 years of follow up or in females at either time point. Similarly Kabagambe et al [44] reported no association between lower albumin concentrations and all-cause mortality (HR 0.81, 0.74–0.89) and Baylis et al [42] found no association in males or females at 10 years follow up (HR 0.93, 0.77–1.11, p = 0.401).

#### Ferritin

Menke et al [46] found that ferritin concentrations varied widely by gender and female menopause status. However, higher ferritin concentrations were not associated with increased risk of all-cause mortality when comparing the fourth versus the second quartiles in premenopausal women (HR 1.43, 0.63–3.23, p = 0.31), postmenopausal women (HR 1.03, 0.79–1.34, p = 0.95) or men (HR 1.09, 0.82–1.44, p = 0.92).

#### Haemoglobin

Baylis et al [42] reported no association between haemoglobin and all-cause mortality risk at 10 years follow up (HR 0.98, 0.78–1.24, p = 0.894).

#### Transthyretin (TTR)

Carriere et al examined the association between TTR and all-cause mortality at two time points. Those participants in the lowest quartile of TTR had a greater risk of mortality at ≤5 years follow up (males HR 2.23, 1.21–4.13, p = 0.01; females HR 2.39, 1.24–4.58, p = 0.009) but this apparently greater risk was not present at 5 to 9 years follow up [30].

#### Cell Adhesion Molecules (CAM). Intercellular Adhesion Molecule-1 (ICAM-1)

Schnabel et al [47] examined the association between inflammatory biomarkers and mortality risk and found a significant positive association between ICAM-1 and mortality risk (HR 1.24, 1.12–1.37, p<0.0001 per 1SD increase in ICAM-1).

#### P-selectin and CD40 ligand

In contrast, Schnabel et al [47] found no associations between P-selectin or CD40 ligand and mortality risk (HR 1.10, 0.99–1.23, p = 0.08 and HR 1.00, 0.90–1.12 p = 0.97 respectively).

#### Cholesterol fractions. Total Cholesterol (TC)

Carriere et al [30] divided participants into sex-specific quartiles of TC at baseline and, to investigate possible non-linear associations between TC and subsequent mortality, created three groups (low quartile, middle quartiles, high quartile), and expressed the HR of mortality relative to the middle category. For both sexes, there were tendencies towards greater mortality in the initial 5 years of follow-up for those in the lowest and highest TC quartiles but this effect was significant (HR 2.21, 1.06–4.62, p = 0.03) for the lowest TC quartile in women only. With longer term follow-up (5 to 9 years), there were no significant associations between baseline TC and subsequent mortality. Cui et al [32] reported a positive linear relationship between baseline TC and cardiovascular mortality with greater mortality risk in males with higher baseline TC concentration (RR 2.07, 1.39–3.08). The association between baseline TC and cardiovascular mortality was weaker, and not statistically significant, in females (RR 1.21, 0.68–2.16).

#### High Density Lipoprotein Cholesterol (HDL-C)

HDL-C was negatively associated with all-cause mortality with a significantly lower risk (p = 0.01) in those with the highest baseline HDL-C concentrations (≥1.82 mmol/L; HR 0.7, 0.53–0.93). When stratified by sex, this trend was significant in females (HR 0.63, 0.41, 0.94, p = 0.03) but not males (HR 0.73, 0.50, 1.06, p = 0.10) [39]. This negative association was also present for cardiovascular mortality (males RR 0.41 (0.27–0.61; females RR 0.34 (0.20–0.57)) [32].

#### Low Density Lipoprotein Cholesterol (LDL-C)

Risk of all-cause mortality was higher in those males and females with the highest baseline LDL-C concentrations (≥4.91 mmol/L;) compared with the lowest concentrations (<3.36 mmol/L; males RR 1.23, 0.96–1.58; females RR 1.12, 0.84–1.49). A similar pattern was seen for cardiovascular mortality (males RR 1.77, 1.22–2.59; females RR 1.37, 0.82–2.27) [32].

#### Non High Density Lipoprotein Cholesterol (Non HDL-C)

There was a positive association between non HDL-C and all-cause mortality for both males (RR 1.49, 1.18–1.88) and females (RR 1.61, 1.22–2.12). Cardiovascular mortality risk was also increased with higher non HDL-C concentrations in both sexes (males RR 2.14, 1.50–3.04; females RR 2.43, 1.47–4.00) [32].

#### Comparison of cholesterol fractions

Cui et al [32] compared the ability HDL-C, non HDL-C and LDL-C to predict CVD mortality risk based on the magnitude of the corresponding RR estimate. In males HDL-C (RR 0.77, 0.69–0.86) and non HDL-C (RR 1.19, 1.13–1.26) were similarly predictive while LDL-C was less predictive (RR 1.11, 1.02–1.22). In females HDL-C was the strongest predictor (RR 0.77, 0.69–0.88) followed by non HDL-C (RR 1.15, 1.06–1.25) and then LDL-C (RR 1.08, 0.96–1.22).

#### Cystatin C (CysC)

Wu et al [51] divided their participants into groups based on coronary risk scores. In males cysC was significantly associated with all-cause and non-CV mortality in the low risk group (HR2.31, 1.42–3.76, p = 0.001; HR 2.32, 1.28–4.19, p = 0.005 respectively), all-cause, CV and non-CV mortality in the intermediate risk group (HR 1.44, 1.10–1.88, p = 0.008; HR 1.60, 1.05–2.44, p = 0.029; HR 1.36, 0.96–1.94, p = 0.084 respectively) and all-cause and CV mortality in the intermediate-to-high risk group (HR 1.22, 1.06–1.40, p = 0.006; HR 1.36, 1.11–1.68, p = 0.004 respectively). In females, cysC was significantly associated with all-cause and CV mortality risk in the intermediate (HR 1.34, 1.09–1.64, p = 0.005; HR 1.73, 1.28–2.33, p<0.001 respectively) and intermediate-to-high risk groups (HR 1.29, 1.08–1.54, p = 0.006; HR 1.52, 1.17–1.98, p = 0.002 respectively).

#### Cytokines and cytokine receptors

In addition to IL-6, Baune et al [31] investigated a range of cytokines including interleukin 1b (IL-1β), interleukin 4 soluble receptor (IL-4sR), interleukin 8 (IL-8), interleukin 10 (IL-10), interleukin 12 (IL-12) and tumour necrosis factor alpha (TNF-α). Baseline concentrations of IL-1β, IL-8, IL-10 and TNF-α were significantly higher in those who died during follow up but, after adjusting for likely confounders, this effect remained significant for IL-8 (p = 0.041) and IL-10 only (p = 0.018). However, in contrast with IL-6, there was no dose-response relationship between tertiles of IL-8 (HR 1.35, 0.7–2.50, p = 0.34) and IL-10 (HR 1.24, 0.7–2.1, p = 0.45 and mortality risk. Baylis et al [42] found weak evidence that IL-1β was associated with all-cause mortality (HR 1.17, 1.00–1.36, p = 0.044) since this relationship was significant in the unadjusted model only. Schnabel et al [47] found no association between monocyte chemoattractant protein-1 (MCP1) and mortality.

#### Enzymes

There were no associations between matrix metalloproteinase 9 (MMP-9) (HR 1.24, 0.62–2.49, p = 0.54) [48] or myeloperoxidase (HR 1.02, 0.91–1.13, p = 0.78) [47] and mortality risk. Whilst lipoprotein associated phospholipase A2 (Lp-PLA2) activity was associated with mortality risk (HR 1.16, 1.01–1.32, p = 0.03) Lp-PLA2 mass was not (HR 1.07, 0.96–1.19, p = 0.20) [47].

#### Erythrocyte Sedimentation Rate (ESR)

Baylis et al [42] reported a significant association between higher ESR and increased mortality risk (HR 1.33, 1.11–1.58, p = 0.002).

#### Glycoproteins. Alpha 1-acid glycoprotein (AAG)

Carriere et al (2008) reported that AAG concentrations in the highest quartile were associated with significantly increased mortality risk at ≤5 years follow up (males HR 2.26, 1.19–4.31, p = 0.01; females HR 2.61, 1.27–5.35, p = 0.009). At 5 to 9 years follow up, compared with the middle quartiles, mortality rates for men in the lowest quartile were reduced significantly (HR 0.38, (0.16–0.92, p = 0.03) but there was no such effect in women [30].

#### Fibrinogen

Wu [51] reported a significant association between fibrinogen and all-cause mortality (HR1.43, 1.10–1.88, p = 0.009 and cardiovascular mortality (HR1.69, 1.13–2.52, p = 0.01) in males with intermediate cardiovascular risk. Schnabel et al [47] also found that higher fibrinogen concentrations were associated with increased all-cause mortality risk (HR 1.15, 1.03–1.29, p = 0.02) in males and females combined.

#### Tissue Inhibitors of Metalloproteinases 1 (TIMP1)

Higher levels of TIMP-1 were associated with greater risk of mortality (HR 1.97, 1.53–2.53, p = 0.001) [48].

#### Sex Hormone Binding Globulin (SHBG)

There was no evidence that SHBG levels were associated with mortality risk (HR 0.93, 0.77–1.11, p = 0.411) [42].

#### Transferrin

Menke et al [46] reported that levels of transferrin saturation differed by gender and menopause status. However, there was no evidence that higher levels of transferrin were associated with increased risk of all-cause mortality when comparing the fourth versus the second quartiles in premenopausal women (HR 1.48, 0.70–3.11, p = 0.60), postmenopausal women (HR 1.17, 0.92–1.49, p = 0.63) or men (HR 1.08, 0.82–1.43, p = 0.62).

#### Homocysteine

Among American participants in the Third National Health and Nutrition Examination Survey (NHANES III), higher plasma homocysteine concentrations were associated with increasing CV mortality risk (HR 1.30, 1.02–1.66, p = 0.032) [51].

#### Hormones

Baylis et al [42] found no associations between mortality risk and concentrations of cortisol (HR 1.08, 0.90–1.31, p = 0.409), testosterone (in males only; HR1.18, 0.98–1.42, p = 0.075), thyroid stimulating hormone (TSH) (HR 0.94, 0.78–1.13, p = 0.521) or thyroxine (T4) (HR 0.95, 0.79–1.14, p = 0.609).

#### Inflammation-related Receptors. Osteoprotegerin (OPG) and Receptor Activator of Nuclear Factor KappaB ligand (RANKL)

Among 3250 Framingham Study participants (54% of whom were women), there was a positive association between OPG and all-cause mortality (HR 1.31, 1.14–1.50, p = 0.0002) [45]. However Lieb et al [45] found no association between RANKL and mortality risk (HR 0.89, 0.78–1.01, p = 0.07) in the same study.

#### Soluble Urokinase Plasminogen Activator Receptor (suPAR)

Higher baseline concentrations of suPAR were associated with increased risk of mortality at 13 years of follow up. Males in the lowest suPAR quartile at baseline survived on average 8.4 years longer than those in the highest quartile (p<.0001) while females in the lowest quartile survived an average of 4.7 years longer (p<.0001) [33].

#### Tumour Necrosis Factor Receptor II (TNFRII)

In a community-based study of 3035 participants, Schnabel et al [47] reported a significant association between TNFRII and mortality risk (HR 1.33, 1.19–1.49, p = <0.0001).

#### Metabolites. Creatinine

Serum creatinine was predictive of CV mortality only in intermediate and high coronary risk groups among NHANES III participants [51].

#### Dehydroepiandrosterone Sulphate (DHEAS)

In community-dwelling older people, there was no association between DHEAS and all-cause mortality (HR 1.18, 0.97–1.43, p = 0.091), although lower levels of DHEAS were associated with increased risk of frailty at ten years follow up [42].

#### Serum Uric Acid (SUA)

Three studies investigated the relationship between SUA and mortality and found similar effects [38, 40, 51]. Using data from 1423 middle-aged Finnish men, Niskanen et al [38] reported an increase in all-cause mortality risk between the lowest (3.03–5.08 mg/dL) and highest (5.89–9.58 mg/dL) tertiles of baseline SUA concentrations (RR 1.82–1.12–2.97, p = 0.02) and cardiovascular mortality risk was greater in those with the highest SUA concentrations (RR 3.73, 1.42–9.83, p = 0.01). Wu et al [51] also reported a significant association between SUA and all-cause mortality in male participants in NHANES III with low CV risk (HR 1.15, 1.04–1.27, p = 0.007). In a large cohort of 28,613 Austrian women, Strasak et al [40] reported greater risk of cardiovascular mortality in those in the highest versus the lowest quartiles of SUA (HR 1.52, 1.37–1.70; p<0.0001).

### Procollagen Type III Aminoterminal Peptide (PIIINP)

For 922 Framingham Study participants, each SD increase in log-PIIINP was associated with almost 50% increased mortality risk (HR 1.48, 1.13–1.93, p = 0.004) [48].

### White Blood Cell (WBC) Individual Components

Two reported associations between WBC components and mortality. Among almost 10,000 Korean elders, all-cause mortality risk increased significantly in those with the highest compared with the lowest quartiles of granulocytes (HR 3.29, 1.87–5.78, p<0.001) [41]. Among participants of the Hertfordshire Ageing Study, higher neutrophils were associated with increased mortality (HR 1.33, 1.11–1.59, p = 0.002) [42]. The results for monocytes were less consistent. Kim et al [41] reported higher mortality risk for those in the highest versus the lowest quartiles of monocytes (HR 9.93, 4.78–20.65, p<0.001) whereas Baylis et al [42] found no significant association (HR1.19, 1.00–1.43, p = 0.054). The evidence for links between lymphocyte count and mortality was weak. Baylis et al [42] found no association with mortality risk (HR 1.10, 0.91–1.32, p = 0.319) while Kim et al [41] reported a significant association (HR 0.68, 0.42–1.08, p = 0.006) which was lost in the adjusted models.

## Discussion

### Summary of principal findings

This systematic review identified 51 blood-borne biomarkers for which relationships between baseline values and subsequent mortality risk were assessed in individuals initially aged 50–75years. The biological role of each of these biomarkers is described briefly in Table 2. Of these 51 potential biomarkers, there was evidence of significant associations with mortality risk for 20 biomarkers. The strongest evidence available was for those biomarkers for which there were sufficient studies to allow meta-analyses. These meta-analyses confirmed that higher baseline concentrations of CRP, NT-proBNP and WBC count were associated with greater mortality risk. There was also more limited evidence that BNP, cholesterol fractions (TC, non HDL-C, HDL-C & LDL-C), ESR, fibrinogen, granulocytes, homocysteine, ICAM-1, neutrophils, OPG, PIIINP, SUA, SUPAR, TIMP1 and TNFRII also predicted mortality. Relationships with mortality for AAG, albumin, creatinine, cysC, IL-1β, IL-4sR, IL-8, IL-10, LpPLA2, lymphocytes, monocytes, OH(25)D, TNFα and TTR were less consistent and did not remain stable between studies, over time of follow up, between participant groups or after adjusting for confounders. There was no evidence of significant associations between baseline CD40, cortisol, DHEAS, ferritin, haemoglobin, IL-12, MCP1, MMP-9, myelopereoxidase, P-selectin, RANKL, SHBG, T4, testosterone, transferrin, and TSH and mortality risk.

**Table 2:** A brief explanation of the role of each biomarker.

Biomarker	Role
**25 Hydroxyvitamin D (25(OH)D)**	Major circulating metabolite of vitamin D used as a biomarker of vitamin D status [51]
**Brain Natriuretic Peptide**	Brain Natriuretic Peptide (BNP) is a cardiac hormone predictive of cardiovascular events which is secreted from cardiomyocytes together with N-Terminal pro-Brain Natriuretic Peptide (NTproBNP) which is biologically inactive but has a longer half-life [37].
**Carrier Proteins**	Albumin is associated with inflammation [44]. Ferritin reflects levels of boy iron stores [46]. Haemoglobin transports oxygen in red blood cells and Transthyretin (TTR) is a carrier of thyroxine and a marker of nutritional status [30].
**Cell Adhesion Molecules**	Intercellular Adhesion Molecule-1 (ICAM-1), P-selectin and CD40-ligand are all markers of intercellular adhesion [47].
**Cholesterol Fractions**	Total Cholesterol (TC), High Density Lipoprotein Cholesterol (HDL-C) and Low Density Lipoprotein Cholesterol (LDL-C) are atherogenic and can reflect risk of cardiovascular disease [32]. Non High Density Lipoprotein Cholesterol (non HDL-C is the difference between TC and HDL-C concentration and contains all of the known, potentially atherogenic lipid particles [30, 32].
**Cystatin C**	Cystatin C is a marker of glomerular filtration rate and predictive of risk of cardiovascular events [51].
**Cytokines/Cytokine Receptors**	Interleukin 1b (IL-1b), Interleukin 4 Soluble Receptor (IL-4sr), Interleukin 8, (IL-8), Interleukin 10 (IL-10), Interleukin 12 (IL-12) and Tumour Necrosis Factor Alpha (TNFa) are cytokines which are markers of inflammatory response [31]. Monocyte Chemoattractant Protein-1 (MCP1) is a chemokine involved in inflammatory response [47].
**Enzymes**	Metalloproteinase 9 (MMP-9) is a marker of extra cellular matrix regulation [48]. Myeloperoxidase is an enzyme released during the immune response [47]. Lipoprotein-associated Phospholipase A2 (Lp-PLA2) is a marker of inflammatory response and oxidative stress [47].
**Erythrocytes**	Erythrocyte Sedimentation Rate (ESR) is used as a marker of inflammation [42].
**Glycoproteins**	Alpha 1-acid Glycoprotein (AAG) is an acute phase inflammatory marker [30]. Fibrinogen is marker of renal function [51] and inflammation [47], Tissue Inhibitors of Metalloproteinases (TIMP1), Sex Hormone Binding Globulin (SHBG) is a glycoprotein which binds sex hormones [42]. Transferrin binds iron [46].
**Homocysteine**	Homocysteine is associated with kidney function [51]
**Hormones**	Cortisol is an immunosuppressant produced by the hypothalamic-pituitary axis [42]. Testosterone is a steroid hormone and Thyroid Stimulating Hormone (TSH) is a pituitary hormone [41].
**Inflammatory-related Protein and Receptors**	C-Reactive Proteins (CRP) is an acute-phase protein produced in response to inflammation [30] however there is some debate about the utility of CRP as an independent predictor of mortality risk (e.g. [53, 54, 55]. Osteoprotegerin (OPG) and Receptor Activator of Nuclear Factor KappaB Ligand (RANKL) are involved in bone mass regulation and vascular remodelling [45]. Soluble Urokinase Plasminogen Activator Receptor (suPAR) reflects inflammatory and immune responses [33]. Tumour Necrosis Factor Receptor II (TNFRII) is a cytokine involved in the acute-phase response [47].
**Metabolites**	Creatinine is used as a marker of renal function and is associated with risk of cardiovascular events [51]. Dehydroepiandrosterone Sulphate (DHEAS) is a marker of cardiovascular disease, osteoporosis and mortality risk [42]. Serum Uric Acid (SUA) is a marker of renal function [51] and is controversially related to cardiovascular events [40].
**Procollagen Type III Aminoterminal Peptide (PIIINP)**	PIIINP is a marker of collagen turnover [48].
**White Blood Cells (WBCs)**	Granulocytes, Monocytes, Lymphocytes and Neutrophils are white cells involved in immune response [42], WBC Count is marker of systemic inflammation [41].

### Strengths and limitations

This systematic review is the first to examine the potential utility in predicting mortality risk of blood-borne biomarkers. Whilst a relatively large number of putative risk biomarkers were identified, in most cases there were only a few studies reporting on a given biomarker and these studies differed considerably in terms of participant numbers and characteristics. This limited our ability to undertake meta-analyses and sub-group analyses so caution should be used when interpreting findings from those biomarkers considered in the narrative review. Furthermore, as a sensitivity analysis for CRP where we had the most studies, the meta-analysis was re-run after excluding the data from Makita et al [36], which had the largest sample size. The results remained consistent with the outcomes from original meta-analysis (S1 Fig). The review was constrained by available data, particularly the heterogeneity of mortality in different study populations. In addition the categorisation of high and low levels of a given biomarker was determined by the criteria used by the authors of each as individual level data were not used in this review. A strength of the current analysis is that it focussed on studies which used prospective cohort designs and this identified studies which had relatively large sample sizes and relatively long durations of follow up. A weakness of the evidence included in this review is that that the biomarkers were measured only once at baseline, and therefore there is no information on risk of misclassification and no data about changes in the biomarker over the follow up period. Although the included studies were performed in several locations in Europe, the USA, Japan and Korea, it was not possible to determine whether there were systematic differences in the predictive utility of specific biomarkers among different ethnic groups or according to geography.

### Relationship to previous studies

All of the blood-borne biomarkers identified in this review fulfil some of Johnson’s [14] criteria for biomarkers of ageing because they can be tested repeatedly without harm to the person and, at least in principle, would work equally well in animal models. Some, e.g. those that assess inflammation or metabolic stress, may monitor processes that underlie ageing. However, further work is needed to confirm that these blood-borne biomarkers are a better measure of ageing than chronological age. Since some of the biomarkers (e.g. blood lipids concentrations) are well-established biomarkers of cardiovascular disease risk, it is unlikely that many, if any, of the biomarkers reported in the present study will fulfil the criterion of measuring only ageing processes and not disease processes. In this respect, it is likely to prove difficult to separate biomarkers of ageing from those which predict risk of age-related disease.

### Interpretation and importance of the findings

The main finding of this systematic review was the identification of 20 blood-borne biomarkers that predict mortality risk in middle-aged people. Of these 20, there were sufficient, similar studies reporting on well-established biomarkers such as CRP, NT-proBNP and WBC count to permit meta-analysis which confirmed the potential predictive value of these biomarkers. More evidence is necessary to establish the value of novel biomarkers and whether these add further predictive value to more established ones. One possible use of the biomarkers identified here is as outcome measures in future intervention studies aimed at enhancing healthy ageing. However, it cannot be assumed that blood-borne biomarkers which predict (or are associated with) risk of mortality will be suitable as outcome measures for such intervention studies because healthy ageing has yet to be defined adequately and, as a consequence, surrogate endpoints such as mortality may be inappropriate [12]. In addition, blood-borne biomarkers measured in middle age which are predictive of later health may not be reliable when used with other age groups (e.g. [56]).

### Implications for further research

The blood-borne biomarkers identified in this analysis need to be fully evaluated for their predictive capability and responsiveness to lifestyle-based interventions before they can be recommended to assess the utility of such interventions. In addition, it will be important to know i) whether there is redundancy within the 20 biomarkers identified here and ii) which of the biomarkers are the most predictive. Given that ageing is a complex process affecting all body systems, it is possible that a panel of biomarkers (a subset of the 20 identified by this review) would be better than any of the biomarkers individually; this hypothesis also needs to be tested. It may be possible to compare the utility of a range of biomarkers using data from different study designs using the horizontal systematic review method (e.g. [57]). Expansion of the age range for participants included in the review would be useful to determine whether the most predictive biomarker panels change with age.

## Supporting Information

S1 File Search strategy for Medline, Embase, Web of Science and PsycInfo. (DOCX)

S2 File Data Extraction Form. (DOCX)

S1 Fig Forest plot of CRP meta-analysis with data from Makita et al excluded. (TIF)
