# Supplementary material for: Blood-Borne Biomarkers of Mortality Risk: Systematic Review of Cohort Studies
Source: PLoS One. 2015 Jun 3;10(6):e0127550. doi: 10.1371/journal.pone.0127550 (PMC4454670; doi:10.1371/journal.pone.0127550)
Supplement: S1 File — (DOCX) [file pone.0127550.s001.docx]

| **Strategy** | **Database** | | | |
| --- | --- | --- | --- | --- |
|  | **Medline** | **Embase** | **Web of Science** | **Scopus** |
| **Cohort study filter** | 1. exp cohort studies/ 2. cohort$.tw. 3. controlled clinical trial.pt.  4. epidemiologic methods/ 5. limit 4 to yr=1971-1988 6. or/1-3,5 (a combination of 1 or 2 or 3 or 5). | 1. exp cohort analysis/ 2. exp longitudinal study/ 3. exp prospective study/ 4. exp follow up/ 5. cohort$.tw. 6. or/1-5. | Topic=cohort OR cohort study | Indexterms (cohort OR cohort study) |
| **Biomarker** | Exp biological marker/ OR  marker$.mp | Exp biological marker/ OR  marker$.mp | Topic = biological marker OR marker* | Indexterms (biological marker) OR Key (marker*) |
| **Blood terms** | Blood/ OR  Plasma/ OR  Serum/ OR  DNA/ OR  Circulat$.mp | Blood/ OR  Plasma/ OR  Serum/ OR  DNA/ OR  Circulat$.mp | Topic = (blood OR serum OR plasma OR DNA OR circulat*) | Indexterms (blood OR plasma OR serum OR DNA) OR Key (circulat*) |
| **Age terms** | Aged/ OR middle aged/ | Limit to (adult <18 to 64 years> OR aged <65+years>) | Topic = (aged OR middle aged OR adult) | Indexterms (aged OR middle aged OR adult) |
| **Mortality terms** | Mortality/ OR mortality.mp OR longevity/ OR longevity.mp OR lifespan/ OR lifespan.mp | Mortality/ OR mortality.mp OR longevity/ OR longevity.mp OR lifespan/ OR lifespan.mp | Topic = (mortality OR lifespan OR longevity) | Indexterms (mortality OR lifespan OR longevity) OR Key (mortality OR lifespan OR longevity) |
| **Other** | Limit to (English language and humans) | Limit to (human and English language) | Refine by: Languages=English | Limit to (Language ‘English’) |
