# Supplementary material for: Blood-Borne Biomarkers of Mortality Risk: Systematic Review of Cohort Studies
Source: PLoS One. 2015 Jun 3;10(6):e0127550. doi: 10.1371/journal.pone.0127550 (PMC4454670; doi:10.1371/journal.pone.0127550)
Supplement: S2 File — (DOCX) [file pone.0127550.s002.docx]

| 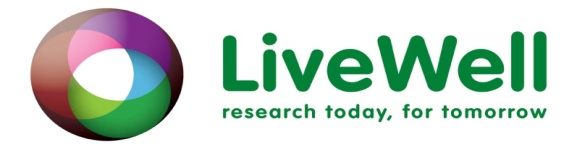 | **Project:**  **Blood-borne Biomarkers** |
| --- | --- |

**Data Extraction Form**

| **Completed by:** | **Date:** |
| --- | --- |

| **General info** | **Guidance** | **Information extracted** | |
| --- | --- | --- | --- |
| Reference | Provide article’s details | **Title:__________________________________________________________**  **______________________________________________________________**  **Journal name:__________________________________________________**  **Year_______ Volume________ Pages________** | |
| First author |  |  | |
| Endnote #ref |  |  | |
| Full article in English language? Yes No | | |  |
| **Cohort Characteristics** | **Guidance** | **Information extracted** | |
| Cohort name |  |  | |
| Cohort location | Country/city |  | |
| Enrolment Period | Year(s) of enrolment |  | |
| No. enrolled |  |  | |
| Follow up period(s) | Date of follow up(s)  Length of follow up period |  | |
| Mean length of follow up |  |  | |
| No. At each follow up | No. of participants at each follow up |  | |
| Rationale of study | What was this study designed to assess? | **Main aim/objective:**  **Secondary aims/objectives:**  **Hypotheses:** | |
| **Participants Characteristics** | **Guidance** | **Data extracted** | |
| Age of participants |  | **Mean: SD: Range:** | |
| Gender | Delete as appropriate | **Male (n/%): Female (n/%):** | |
| Ethnicity | Describe the ethnic background of the sample | **Not stated** | |
| Inclusion criteria |  |  | |
| Exclusion criteria |  |  | |
| Health of the sample | List any disease risk factors and n for each |  | |
| Method of selection | Describe how participants were chosen |  | |
| Socioeconomic status/ Social class |  |  | |
| **Outcomes** | **Guidance** | **Data extracted** | |
| Biomarker measured at baseline or follow up? |  |  | |
| Exposure |  |  | |
| Biomarkers examined | Provide details of biomarkers examined |  | |
| Sample analysed |  | **Whole blood**  **Plasma**  **Serum**  **Other** __________________________________ | |
| Method of assessment |  |  | |
| Measurement of biomarker(s) |  | **Method of measurement:**  **Unit of measurement:** | |
| Comparison of biomarkers to other markers | If yes, list markers | **No**  **Yes**  __________________________________________  __________________________________________ | |
| Mortality | How has mortality been measured? E.g. deaths within a certain time frame or mean lifespan of sample? Give details. |  | |
| Other results | Any other results of not? Describe |  | |
| **Analysis** | **Guidance** | **Data extracted** | |
| Statistical technique(s) used |  |  | |
| Description of withdrawals | Any information provided on withdrawals and subjects lost to follow up? | **Yes No Not clear** | |
| Comparison of non-respondents, withdrawals with completers |  |  | |
| Adjustment for confounders? |  | **Yes No Not clear**  **AND**  **Please list the confounders adjusted for____________________________ ______________________________________________________________** | |
| Format of reported data |  | **Raw data Unadjusted for confounders**  **Adjusted for confounders Unadjusted and adjusted**  **Standardised by sample distribution Adjusted and standardised** | |
| **Summary** | **Guidance** | **Data extracted** | |
| Key results |  |  | |
| Limitations |  |  | |
