# Supplementary figures and images for: Blood-Borne Biomarkers of Mortality Risk: Systematic Review of Cohort Studies
Source: PLoS One. 2015 Jun 3;10(6):e0127550. doi: 10.1371/journal.pone.0127550 (PMC4454670; doi:10.1371/journal.pone.0127550)

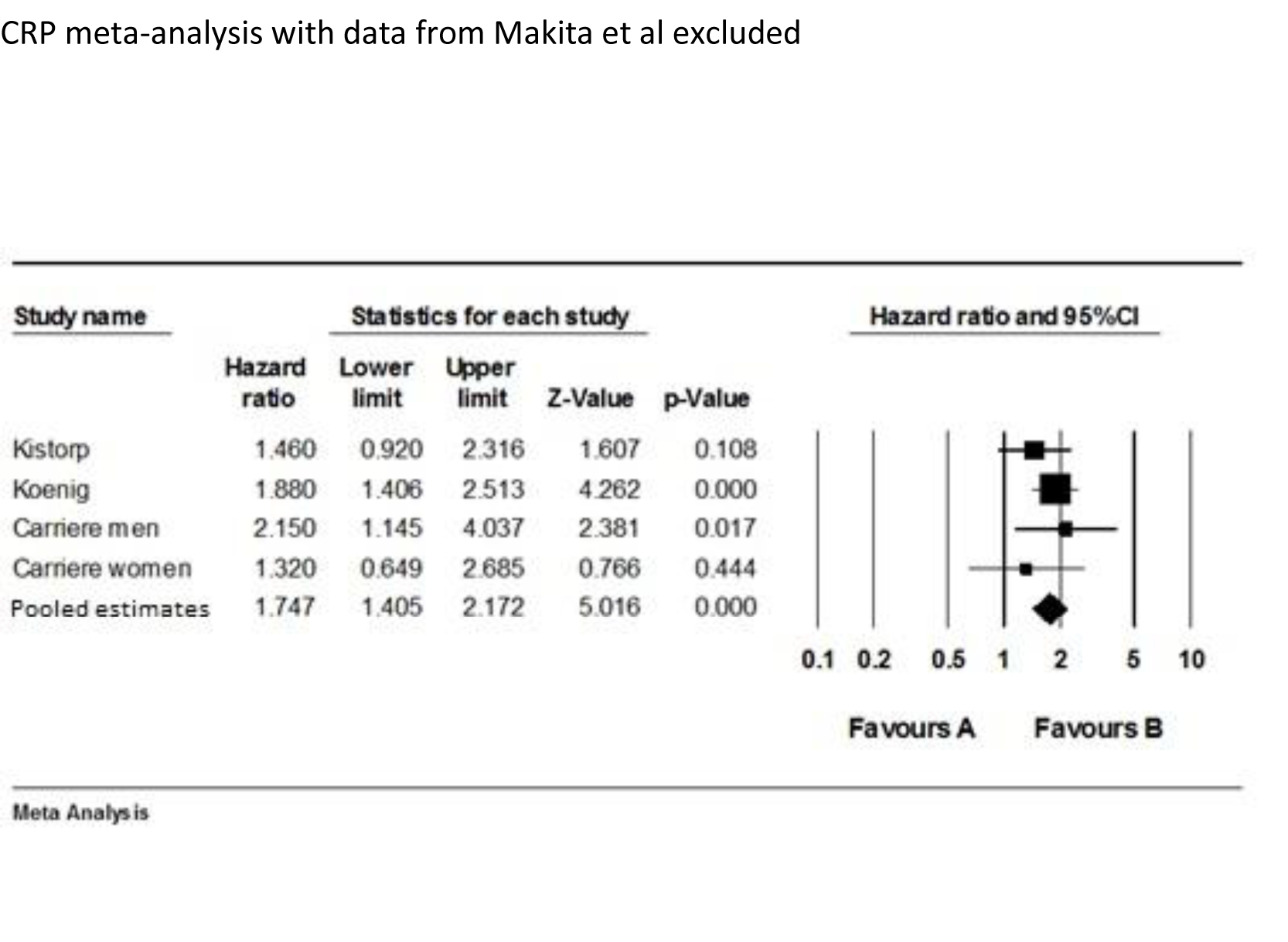

Supplement: S1 Fig — (TIF) [file pone.0127550.s003.tif]
